# Improving depression screening in HIV positive pregnant women

**DOI:** 10.1192/j.eurpsy.2021.1595

**Published:** 2021-08-13

**Authors:** D. Bhullar, S. Gunturu, M. Mirhom

**Affiliations:** 1 Psychiatry, Bronx Care Health System, NYC, United States of America; 2 Psychiatry, Columbia University, NYC, United States of America

**Keywords:** women’s mental health, HIV, Depression, Depression screening

## Abstract

**Introduction:**

Depression is a common complication of pregnancy and the postpartum period. Up to 70% percent of women report depressive symptoms during their pregnancy, and approximately 10-16% meet full criteria for major depressive disorder. Women with a history of perinatal or non-perinatal major depression are likely to relapse during pregnancy. Research shows that exposure to untreated depression and stress can have negative consequences on the birth outcome and child development. Given the harmful effects of this disease on both the mother and child, it is essential that all pregnant patients be screened for depression. Literature review did not reflect many studies that focus on depression screening in this population, let alone in pregnant patients with Human Immunodeficiency Virus (HIV). Our study focuses on the impact the mandatory screening tool had on the incidence of depression screening in pregnant HIV patients.

**Objectives:**

- Gain understanding of the Family Focused HIV Health Care Program for Women - Understand the importance of a mandatory screening tool for depression

**Methods:**

Using standardized Quality Improvement tools Implementation of screening tool in notes & enforcing a hard stop in the medical records

**Results:**

We noted both qualitative & quantitative improvement in depression screening. Qualitatively the screening has been standardized by creating a universal workflow by the inclusion of screening tools (PHQ2 and PHQ9) in Electronic Medical records. Quantitatively there has been a 34.9% improvement in screening by the case managers in the post interventional quarter.

**Conclusions:**

Significant improvement noted in the incidence of depression screening by implementation a mandatory screening tool

